# Evidence that tyrosine hydroxylase does not have enzymatic activity in neurons of the supraoptic nucleus

**DOI:** 10.3389/fendo.2026.1777453

**Published:** 2026-04-28

**Authors:** Tatiana S. Pronina, Dmitry V. Troshev, Anna A. Kolacheva, Vsevolod V. Bogdanov, Michael V. Ugrumov

**Affiliations:** Laboratory of Neural and Neuroendocrine Regulations, Koltzov Institute of Developmental Biology of the Russian Academy of Sciences, Moscow, Russia

**Keywords:** hypothalamus, L-3,4-dihydroxyphenylalanine, mice, neuron, osmotic stress, supraoptic nucleus, tyrosine hydroxylase, vasopressin

## Abstract

**Introduction:**

Although numerous neurons expressing tyrosine hydroxylase (TH), the first enzyme of catecholamine synthesis, were discovered in the supraoptic nucleus (SON) of hypothalamus in animals almost forty years ago, mainly under osmotic stress, the functional significance of TH there remains unclear. Therefore, the aim of this study was to test our hypothesis that TH in these neurons has enzymatic activity capable of converting L-tyrosine to L-3,4-dihydroxyphenylalanine (L-DOPA).

**Methods:**

In this study, 8–10 week old male C57BL/6 mice (n = 77), intact (n = 20), and intraperitoneally injected with 0.9% NaCl (n = 29) or 8.5% NaCl (n = 28) were used. To assess gene expression and synthesis of TH, as well as the hypothetical synthesis of L-DOPA in neurons of the SON, the following methods were used: immunohistochemistry for vasopressin (VP), laser microdissection of individual VP-immunopositive neurons, polymerase chain reaction (PCR), Western blotting, and high-performance liquid chromatography with electrochemical detection of dopamine and L-DOPA.

**Results:**

The genes for TH, a key-protein required for L-DOPA synthesis in dopaminergic neurons, is expressed in neurons of the SON in both intact, control and salt-loaded mice. However, some data suggest that TH in neurons of the SON does not have enzymatic activity. Indeed, in intact mice and in mice with varying degrees of osmotic stress: (i) the SON did not show expression of the gene encoding guanosine triphosphate cyclohydrolase 1, the first enzyme in synthesis of tetrahydrobiopterin, a cofactor responsible for regulating the enzymatic activity of TH, and (ii) the amount of L-DOPA remained at the same very low level, regardless of the TH content in SON neurons. We suggest that TH in neurons of the SON does not have enzymatic activity, since, as we have shown, the TH molecule in the SON, unlike TH in dopaminergic neurons of the substantia nigra, could possibly not have an N-terminus motif responsible for regulation of its enzymatic activity.

**Conclusion:**

Despite the expression of the TH gene in neurons of the SON, the protein it encodes does not have enzymatic activity, most probably due to the absence of the N-terminus, characteristic of TH in dopaminergic neurons.

## 1 Introduction

The discovery in the last century of large secretory neurons, known as magnocellular neurons (1, 2), first in the anterior hypothalamus of fish, and then in the supraoptic nucleus (SON), paraventricular nucleus and the accessory nuclei of the hypothalamus in rats, was the trigger for the development of a new paradigm of integrative physiology – neuroendocrinology (3). Over time, it has been shown that the secretory products of these neurons are vasopressin (VP), oxytocin, or both neuropeptides simultaneously (4–6).

The axons of magnocellular neurons project mainly into the pituitary posterior lobe, where VP and oxytocin are released into the general circulation due to the absence of the blood-brain barrier, acting as neurohormones on peripheral target organs. VP is involved in the regulation of: (i) water-salt metabolism, exerting an antidiuretic effect on the kidneys, (ii) the cardiovascular system, inducing vasoconstriction, (iii) glucose metabolism in the liver (7–10). The main physiological effect of oxytocin is to stimulate contraction of the smooth muscles of the uterus and mammary glands (9, 10).

Since the 1980s, it has been repeatedly shown in various mammals that some VPergic and oxytocinergic neurons co-express tyrosine hydroxylase (TH) (11, 12), the first enzyme of catecholamine synthesis in catecholaminergic neurons (13, 14). TH expression in these neurons is species-specific. In most rodents, TH is detectable by immunological methods only under certain functional conditions (osmotic stimulation, hypoxia, etc.) (11, 15–19). However, in some mammals, TH is co-expressed in magnocellular neurons under both normal and pathological conditions (20–24).

When studying magnocellular neurons, researchers came to the same conclusion: TH plays an important role in the functioning of these neurons. All attempts to elucidate the functional significance of TH so far have been unsuccessful. Nevertheless, many authors proceed from the idea that TH in magnocellular neurons, as in catecholaminergic neurons, is an enzyme that converts L-tyrosine to L-3,4-dihydroxyphenylalanine (L-DOPA). However, until now no convincing evidence for this has been obtained (12). The only indirect argument in favor of the enzymatic activity of TH is considered to be the expression of the guanosine triphosphate cyclohydrolase 1 (GCH1) gene in human magnocellular neurons (24), an enzyme involved in the synthesis of tetrahydrobiopterin, co-factor for TH (25). Meantime, this enzyme is not detectable by immunological methods in magnocellular neurons in either humans (25) or rodents (17).

Advances in immunohistochemistry and molecular biology techniques, as well as the development of new models with high levels of TH expression in hypothalamic magnocellular neurons over recent years (26), have made it possible to return to testing the role of TH in these neurons. Therefore, the aim of this study has been to test the hypothesis that TH in neurons of the SON is the enzyme that converts L-tyrosine to L-DOPA. To meet this goal, the following objectives have been addressed:

1. To determine whether, in addition to the VP gene, the TH and GCH1 genes are expressed in neurons of the SON in intact and osmotically stimulated mice;
2. To determine whether the content of L-DOPA, as a product of the enzymatic activity of TH in catecholaminergic neurons, changes in the SON during osmotic stimulation of animals compared with the L-DOPA content of intact animals;
3. To analyze the structure of TH in VP neurons of the SON.

## 2 Materials and methods

### 2.1 Animals

Male C57BL/6 mice (n = 77) aged 8–10 weeks and weighing 22–25 g, obtained from the Stolbovaya breeding center (SCBMT RAMS, Stolbovaya, Moscow region, Russia), were used in the study. The animals were kept at 22 ± 1 °C, with a 12-h day/night cycle and free access to food and water. The experimental procedures were performed in accordance with the National Institutes of Health Guide for the Care and Use of Laboratory Animals (8th edition, 2011) and were approved by the Animal Care and Use Committee of the Koltzov Institute of Developmental Biology of the Russian Academy of Sciences (protocol No. 85 from 05.09.2024).

### 2.2 Experimental procedures

**Figure 1:**
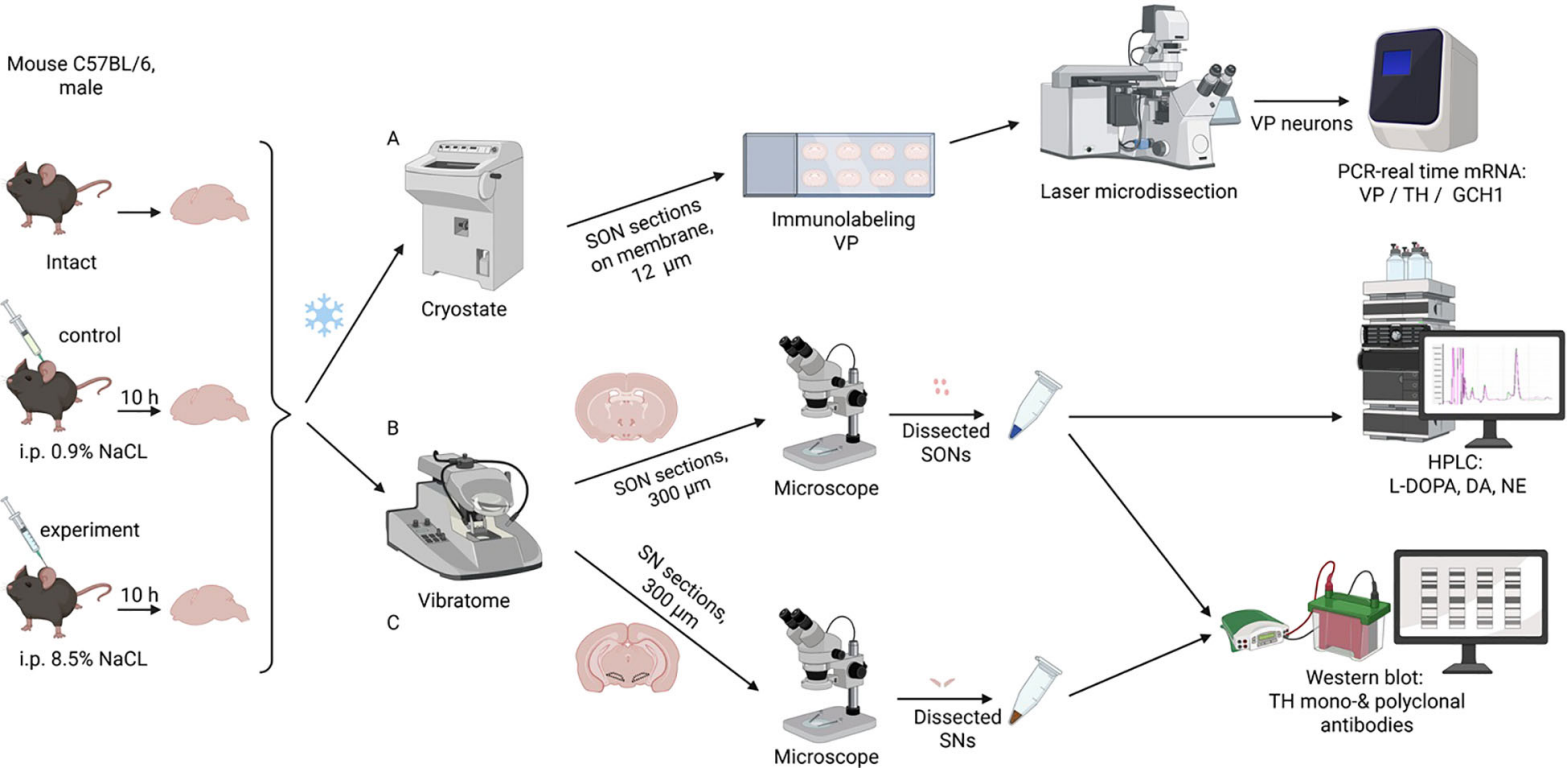
Schematic representation of this study in C57BL/6 mice, intact and 10 hours after intraperitoneal administration of 0.9% NaCl or 8.5% NaCl (left side), including processing of the supraoptic nucleus (SON) as follows: **(A)** Laser microdissection of vasopressin (VP)-immunopositive neuron from 12 μm cryostat sections with subsequent polymerase chain reaction (PCR) of VP, tyrosine hydroxylase (TH), and guanosine triphosphate cyclohydrolase 1 (GCH1) genes; **(B)** Preparation of 300 μm vibratome sections of SON with subsequent assessing L-3,4-dihydroxyphenylalanine (L-DOPA), dopamine (DA), and norepinephrine (NE) or **(C)** Detection of TH in the SON and substantia nigra by Western blot using monoclonal and polyclonal antibodies. Blue asterisk – brain freezing.

Intact mice (n = 20) and mice after a single intraperitoneal administration of 0.9% NaCl (n = 29) or 8.5% NaCl (n = 28) were used in this study (**Figure 1**). They were maintained for 10 h at 22 ± 1 °C, with a 12 h day/night cycle and with free access to food, but without access to water. The animals were then decapitated under anesthesia with 2.4% isoflurane (Laboratorios Karizoo, Spain) using an anesthesia device (SomnoSuite, Kent Scientific, USA). The brains were removed from the skull and processed for analysis using different technical approaches.

### 2.3 Preparation and assessment of brain samples using various methods.

#### 2.3.1 Preparation of supraoptic nuclei and substantia nigra samples for assessing tyrosine hydroxylase content by western blot

The SONs were dissected from frontal vibratome sections of the brains of intact mice (n = 6) and mice pretreated with 0.9% NaCl (n = 11) or 8.5% NaCl (n = 12), as well as in the substantia nigra of intact mice (n = 2), in Krebs-Ringer solution (mM: NaCl 120, KCl 4.8, CaCl2 2, MgSO4 1.3, NaHCO3 25, d-glucose 10, HEPES 20, and ascorbic acid 0.1, pH 7.2; (all reagents for electrophoresis and western blotting were obtained from Sigma-Aldrich, St. Louis, MO, USA, unless otherwise noted)) (**Figure 1**). Substantia nigra was isolated in the same way, but from frontal brain sections of intact mice only. The brain samples were then homogenized in 90 μL RIPA buffer (50 mM Tris base, 150 mM NaCl, 0.1% sodium dodecyl sulfate and 1% Nonidet P-40) and were centrifuged for 20 min at 20,000 x g at 4 °C. Protein concentration was then determined then using Bicinchoninic Acid Solution, a protein assay test (27). After that, proteins were denatured for 5 min at 95 °C in 5% ß-mercaptoethanol. Before sample loading, Laemmli sample buffer containing 2% sodium dodecyl sulfate, 10% glycerol, 5% ß-mercaptoethanol, 62.5 mM Tris (pH 6.8), and 0.004% bromophenol blue was added to each sample it 1:4 volume ratio. 15 μg of protein from SON or 1 to 15 μg of protein from substantia nigra samples were loaded per lane. Electrophoresis was performed on a 12% polyacrylamide gel, after which proteins were transferred to a nitrocellulose membrane (Hybond-enhanced chemiluminescence, Amersham Biosciences, Slough, UK) and maintained overnight at 30 mA. Protein loading and uniform transfer to the membrane were confirmed by Ponceau-S staining (28, 29). Ponceau-S was then removed by incubating the membranes in Tris-buffered saline (100 mM Tris-HCl, 150 mM NaCl and 0.05% Tween 20) with shaking for 10 min. Each membrane was divided into two parts transversely at the level of proteins with a molecular weight of 50–55 kDa. Nonspecific reaction on the membrane was blocked by incubating it in Tris-buffered saline with 5% bovine serum albumin for 1 h at 20 °C with shaking (Sigma-Aldrich, Saint Louis, MO, USA). To determine TH composition in SON the upper parts of the membranes were used. The membranes containing the samples were incubated with primary polyclonal antibodies to TH (1:1000) (AB 1542, Millipore, Burlington, Massachusetts USA) in Tris-buffered saline with 1% bovine serum albumin overnight at 4 °C, or monoclonal antibodies to the N-terminus of TH (1:1000, T1299, Sigma-Aldrich, Saint Louis, MO, USA) under the same conditions. The lower parts of the membranes were incubated with β-actin antibodies (1:5000; A1978, Sigma-Aldrich, Saint Louis, MO, USA). After washing in Tris-buffered saline to remove primary antibodies, the membranes were incubated with secondary antibodies conjugated to horseradish peroxidase for 2 h at 20 °C. Clarity Max Western ECL Substrate (Biorad, Hercules, CA, USA) was used to visualize TH according to the manufacturer’s recommendations.

The chemiluminescence of the bands was recorded with a ChemiDoc Touch device (Biorad, Hercules, CA, USA) for 1–2 min. The chemiluminescence intensity was then measured densitometrically using ImageLab software (Biorad, Hercules, CA, USA). TH signals were normalized to β-actin. To ensure uniform scaling of the data across all membranes, additional normalization was performed using a substantia nigra, which was applied to all membranes.

#### 2.3.2 Preparation of supraoptic nucleus samples and laser microdissection of vasopressin-immunopositve neurons

**Preparation of supraoptic nucleus samples** for laser microdissection (LMD) of VP-immunopositive neurons began with freezing the brain of intact mice (n = 6) and mice pretreated with 0.9% NaCl (n = 6) or 8.5% NaCl (n = 6) in liquid nitrogen vapor according to a previously published protocol (30) (**Figure 1**). 12-µm-thick serial frontal brain sections of 12 µm thickness were then cut in a cryostat (Leica CM1950, Germany) at the level of the SON. The cryostat chamber and instruments were pretreated with 6% hydrogen peroxide and 96% ethanol and were irradiated with UV light for 2.5 hours. The SON sections were mounted onto polyester membranes with 0.9-μm pores (Frame Slides, Leica, Germany), which had been pretreated for 5 min with RNAseClean solution (BioinnLabs, Russia), washed twice with diethylpyrocarbonate -treated water (Evrogen, Russia) for 30 seconds each, washed twice with 96° ethanol for 30 seconds each, and dried in a laminar flow hood under UV light for 45 min. The treated polyester membranes had been stored in a closed dry chamber at 20 °C until section mounting.

**Figure 2:**
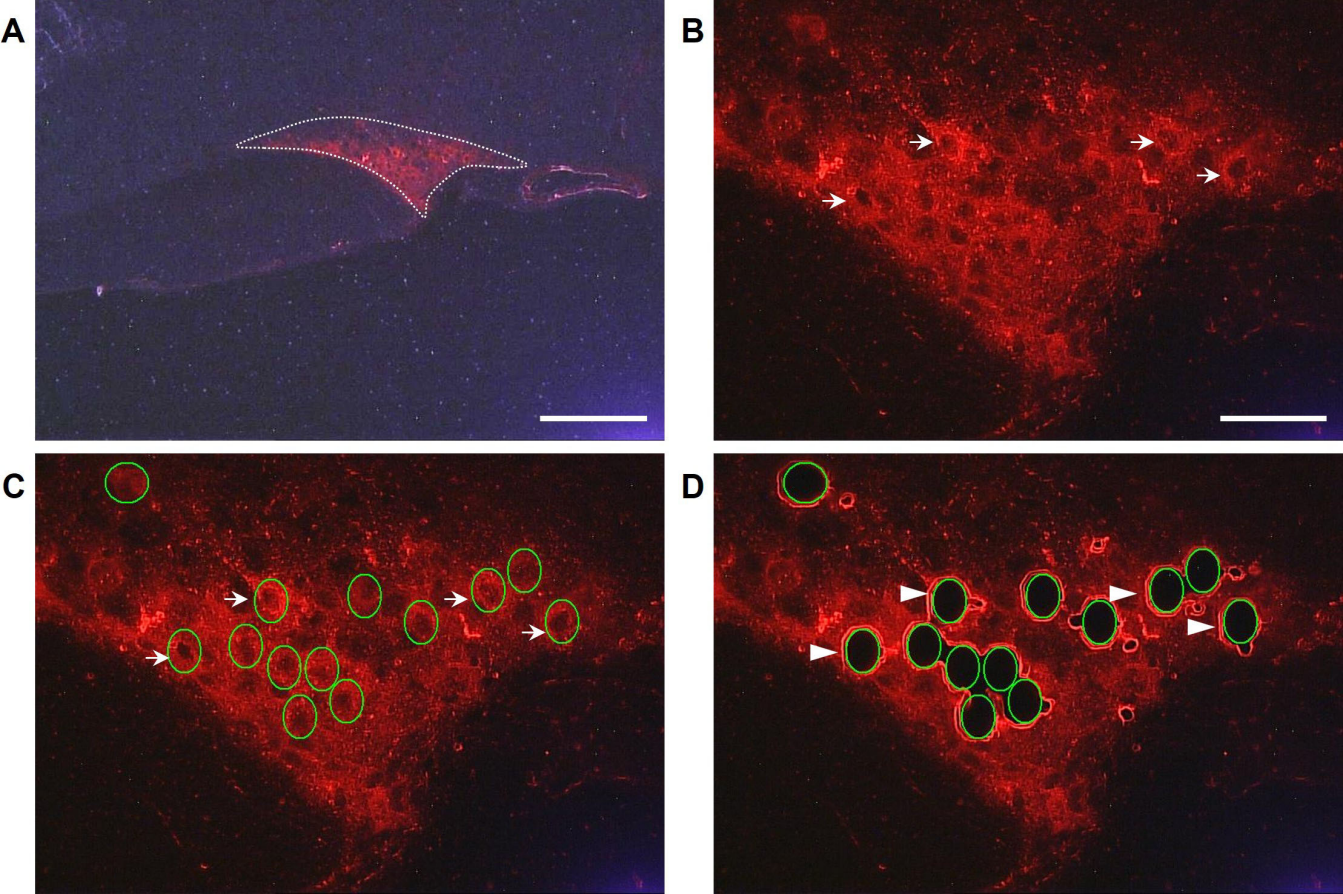
The supraoptic nucleus (SON) of intact 8- to 10-week-old C57BL/6 mice, containing numerous vasopressin-immunopositive neurons [**(A–C)**, red] and holes in their place after laser microdissection [**(D)**, black]. Dotted line – supraoptic nucleus **(A)**; arrows – vasopressin-immunopositive neurons **(B)** and vasopressin-immunopositive neurons indicated by the green line **(C)**; arrowheads – holes after laser dissection of neurons **(D)**. Scale bars: **(A)** – 50 μm, **(B–D)** – 200 μm.

The sections on membranes were fixed in Carnoy’s solution, which included 60% ethanol, 30% chloroform (Sisco, India), and 10% glacial acetic acid (Panreac, Spain), for 3 min at -20 °C. The sections were rinsed sequentially as follows: twice for 30 seconds in 96° ethanol, twice for 30 seconds in 70° ethanol, and twice for 1 min in 0.02 M phosphate-buffered saline containing 1 U/μL RNase inhibitor (SUPERase-in, Invitrogen, Thermo Fisher Scientific, USA) The sections were then incubated with rabbit antibodies to VP conjugated to Alexa Fluor 555 (Abcam, USA) (1:200) in 0.02 M phosphate-buffered saline containing 1 U/μL SUPERase for 75 min at 20 °C. Next, the sections were washed in 0.02 M phosphate-buffered saline containing 1 U/μL SUPERase three times for 1 minute each and were dehydrated with 96° ethanol twice for 30 seconds each. All the solutions were prepared using diethylpyrocarbonate-treated water. Finally, the sections were air-dried for 15 min and were then used for LMD of VP-immunopositive neurons (**Figures 2A–D**).

VP-immunopositive neurons of the SON (**Figures 2A–C**) were dissected using a Leica 7000 microdissector (Germany) at a 40x microscope magnification, laser power 35, laser aperture of 7, speed of 20, and pulse frequency of 1045 (**Figure 2D**). **Figure 2** shows the SON before and after LMD of VP-immunopositive neurons. These neurons were also excised from the SON of mice after intraperitoneal administration of 0.9% or 8.5% NaCl. Laser-dissected VP-immunopositive neurons were collected into 0.6 mL Eppendorf tube caps contained 50 μL of TRI-reagent (MRC, Inc., USA). Each neuronal sample prepared by LMD included 1200 VP-immunopositive neurons. The samples were frozen in liquid nitrogen and stored at -70 °C until mRNA detection by PCR.

### 2.4 Research methods

#### 2.4.1 High-performance liquid chromatography with electrochemical detection

Using high-performance liquid chromatography with electrochemical detection, the concentrations of L-DOPA, dopamine, and norepinephrine were determined in SON samples isolated from the frontal brain sections of intact mice (n = 6) and from those of mice 10 hours after intraperitoneal injection of 0.9% NaCl (n = 12) or 8.5% NaCl (n = 10) (**Figure 1**).

The SON samples were homogenized using a Labsonic M ultrasonic homogenizer (Sartorius, Göttingen, France) in 120 μL 0.1 N HClO_4_ (Sigma-Aldrich, St. Louis, MO, USA) containing 50 pmol/mL of the internal standard, 3,4-dihydroxybenzylamine hydrobromide (Sigma-Aldrich, St. Louis, MO, USA). The protein concentration in each sample was determined using Bicinchoninic Acid Solution, a protein assay test (27). The homogenate was centrifuged for 20 min at 2000 x g.

L-DOPA, dopamine and norepinephrine were separated on a reversed-phase column ReproSil-Pur, ODS-3, 4x100 mm with a pore diameter of 3 μm (Dr. Majsch, Ammerbuch, Germany) at 30 °C with a mobile phase flow rate of 1 mL/min in an LC-20ADsp liquid chromatograph (Shimadzu Corp., Kyoto, Japan). The mobile phase consisted of 0.1 M citrate-phosphate buffer, 0.3 mM sodium octane sulfonate, 0.1 mM EDTA, and 8% acetonitrile (all reagents from Sigma, USA), pH 2.5. An electrochemical detector Decade II (Antec Scientific, Alphen aan den Rijn, Netherlands) (with a glassy carbon working electrode (+0.85 V) and an Ag/AgCl reference electrode) was used. The peaks of interest and the internal standard were identified by their retention times in the standard solution. The contents of L-DOPA, dopamine and norepinephrine were calculated by the internal standard method using a calibration curve in the LabSolutions program (Shimadzu Corp., Kyoto, Japan). The contents of L-DOPA, dopamine and norepinephrine were normalized to the amount of protein in the samples, which was determined using Bicinchoninic Acid Solution for a protein assay (27).

#### 2.4.2 RNA isolation and PCR

Before RNA extraction, two samples of VP-immunopositive neurons of the SON were pooled into a single sample (100 μL) containing 2400 neurons. Thus, in each group of intact mice and mice pretreated with 0.9% or 8.5% NaCl, there were three samples (n = 3). Each sample was adjusted to 1 mL with TRI reagent. 100 μL of 1-bromo-3-chloropropane (Sigma-Aldrich, St. Louis, MO, USA) was added to each sample, and the resulting solution was incubated for 15 min at 20 °C under vortexing. The aqueous and organic phases were then separated by centrifugation at 21,000×g for 15 min at 4 °C.

The aqueous phase containing RNA was transferred to a tube to which 500 μL of isopropyl alcohol (Sigma-Aldrich, St. Louis, MO, USA) and 2 μL of glycogen (Thermo Fisher Scientific, Waltham, MA, USA) were added. The solution was vortexed and incubated for 10 min at 20 °C, followed by RNA precipitation for 20 min at 21,000 × g and 4 °C. The supernatant was collected and the pellet was washed three times with 1 mL of 80% ethanol and was then centrifuged for 10 min at 21,000 × g and 4 °C. After the last centrifugation, ethanol was removed, the RNA pellet was air-dried for 15 min and then dissolved in 11 μL of RNase- and DNase-free water. The RNA concentration in all samples was measured using a NanoDrop 8000 spectrophotometer (Thermo Fisher Scientific, Waltham, MA, USA).

RNA preservation was assessed by electrophoresis in 1% agarose gel containing ethidium bromide (Thermo Fisher Scientific, Waltham, MA, USA). Complementary DNA was synthesized from 150 ng of total RNA using the Maxima H Minus First Strand cDNA Synthesis Kit (Thermo Fisher Scientific, Waltham, MA, USA) according to the manufacturer’s recommendations. Real-time PCR was then performed using qPCRmix-HS SYBR+LowROX (Evrogen, Russia) in a QuantStudio 12k Flex thermocycler (Applied Biosystems, MA, USA). Finally, oligonucleotide primers (**Table 1**, Evrogen, Russia) were used to determine the expression of the following genes: *Cyc1, Avp, Th*, and *Gch1*. 0.5 μg of complementary DNA was used for PCR. All primers were pre-tested to exclude the formation of non-specific amplification products (**Supplementary Figure 1**).

**Table 1:** Oligonucleotide primers used for PCR.

Gene	Forward primer	Reverse primer
*Cyc1*	GCGGCCAGGGAAGTTGT	GCCAGTGAGCAGGGAAAATAC
*Avp*	CCCAAGAGGCGGCAAGAG	CAGGGCGAGGGCAGGTAG
*Th*	TCAGAGGAGCCCGAGGTC	GGGCGCTGGATACGAGAG
*Gch1*	GGCCGCTTACTCGTCCAT	CTTCACAATCACCATCTCGTCA

Using PCR analysis, we first determined which of the studied genes are expressed in VP-immunopositive SON neurons. For this purpose, the amplified products were separated at 100 V in 1.5% agarose gel (Helicon, Moscow, Russia) containing ethidium bromide. DNA fragments were detected using ChemiDoc Touch (Bio-Rad Laboratories, CA, USA). We then performed a comparative analysis of gene expression levels in VP-immunopositive SON neurons using ΔCt. *Cyc1* was taken as a housekeeping gene.


ΔCt = (Ct(gene) − Ct(Cyc1)

where C(t) is the cycle of the intersection of a curve with a threshold line.

#### 2.4.3 Statistics

Statistical analysis was performed using GraphPadPrism 6.0 (GraphPadSoftware, USA). Data are presented as mean ± standard error of the mean (mean ± SEM). Differences were considered statistically significant at p ≤ 0.05. Before comparing the statistical significance of the obtained results, the normality of the distribution of values within the groups was checked. The paired t-test and the nonparametric Mann-Whitney U-test were used to determine the statistical significance of the obtained results.

## 3 Results

### 3.1 Expression of the vasopressin, tyrosine hydroxylase, and guanosine triphosphate cyclohydrolase 1 genes in vasopressinergic neurons of the supraoptic nucleus in mice, including both intact animals and those after intraperitoneal administration of 0.9% NaCl or 8.5% NaCl

In samples of VP-immunopositive neurons obtained by LMD after Carnoy fixation and subsequent immunohistochemistry, the total RNA concentration was constant in all samples, averaging 15 ng/μL.

**Figure 3:**
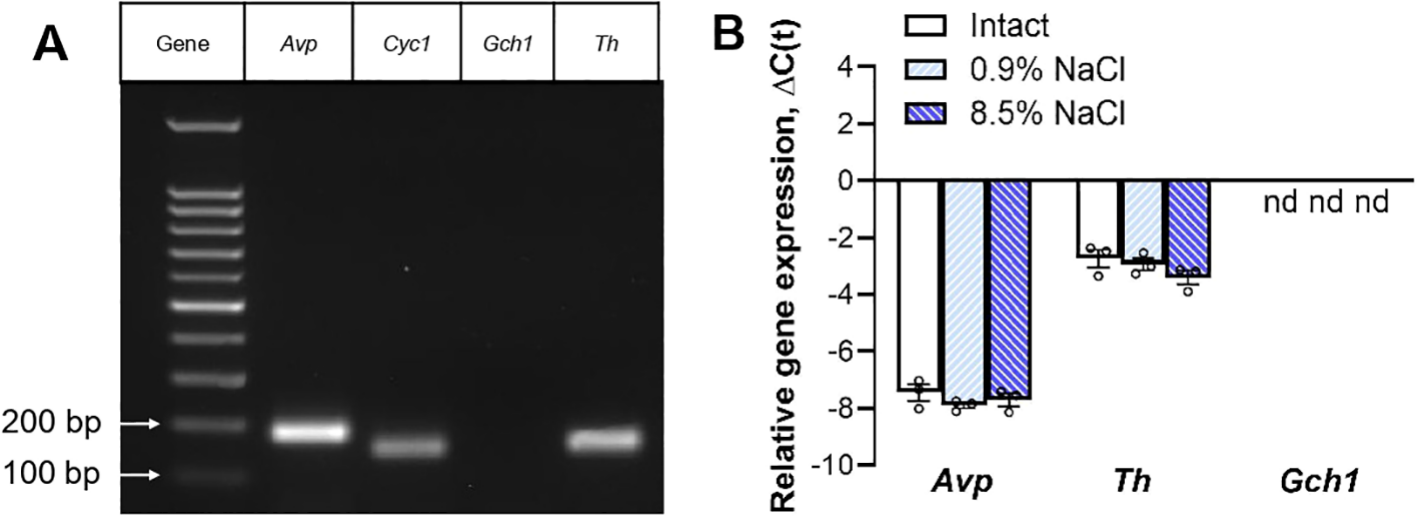
Expression of *Avp*, *Cyc1*, *Th*, and *Gch1* measured by PCR in vasopressin-immunopositive neurons of the supraoptic nucleus obtained by laser microdissection from 8- to 10-week-old C57BL/6 mice: intact (n = 3 samples, 6 animals) and 10 hours after intraperitoneal administration of 0.9% NaCl (n = 3 samples, 6 animals) or 8.5% NaCl (n = 3 samples, 6 animals). **(A)** Agarose gel electrophoresis of PCR products in intact mice. Left lane – DNA ladder (molecular mass marker). bp – base pair. **(B)** Expression levels of *Avp, Th* and *Gch1* represented in ΔC(t) (each sample in 3 technical repeats), nd – not detected. The data are presented as median with interquartile range and individual date (dots).

VP-immunopositive neurons of the SON in intact mice and mice treated with 0.9% NaCl or 8.5% NaCl were shown to express the *Avp* and *Th* (**Figure 3**). *Gch1* expression was not detected in any group (**Figure 3**).

### 3.2 The content of catecholamines and L-DOPA in the supraoptic nucleus in mice, including both intact animals and those after intraperitoneal administration of 0.9% NaCl or 8.5% NaCl

**Figure 4:**
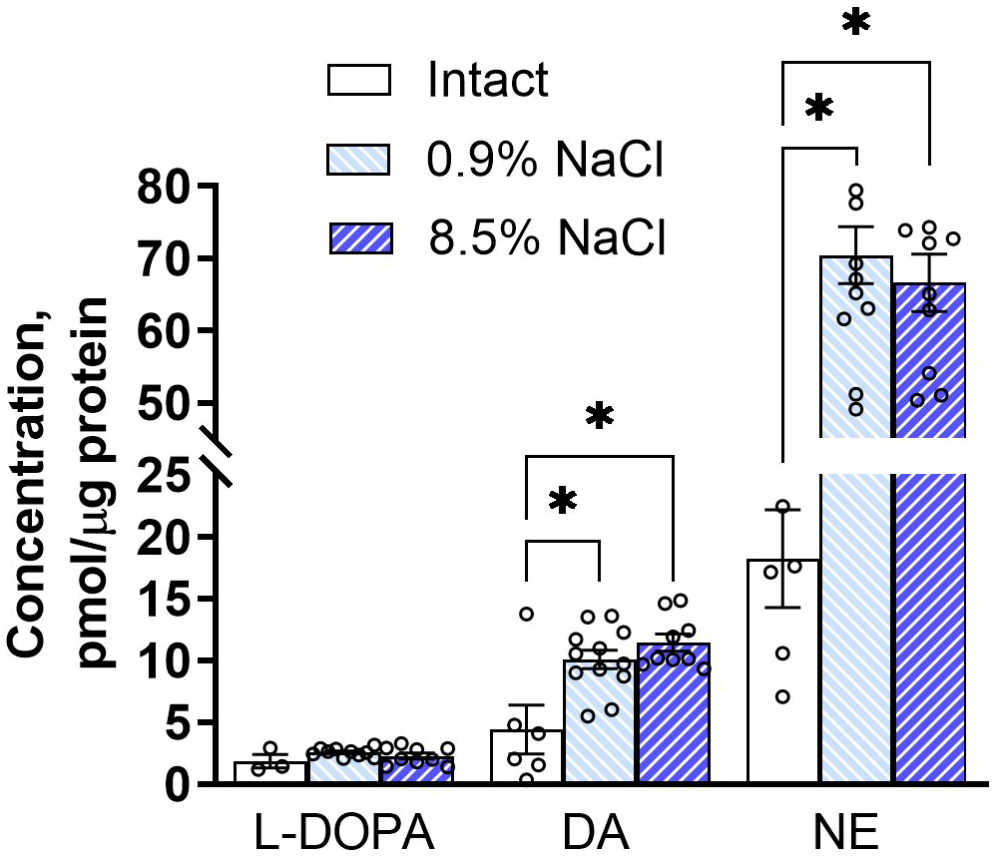
L-3,4-dihydroxyphenylalanine (L-DOPA), dopamine (DA), and norepinephrine (NE) concentrations in the supraoptic nucleus of 8- to 10-week-old C57BL/6 mice, both intact (n = 6) and 10 hours after intraperitoneal administration of 0.9% NaCl (n = 12) or 8.5% NaCl (n = 10), measured by high-performance liquid chromatography with electrochemical detection. Data were normalized for protein content. The data are presented as mean ± SEM with individual date (triangles). Statistical analysis of the data was performed using the Kolmogorov-Smirnov test for normality following the Mann-Whitney or unpaired t-test where applicable. Concentrations of L-DOPA, dopamine (DA), and norepinephrine (NE) were compared between groups of mice: intact and pretreated with 0.9% or 8.5% NaCl. Sample sizes were: 3, 10, and 9 for L-DOPA in intact mice and mice pretreated with 0.9% or 8.5% NaCl, respectively; 6, 12, and 9 for DA in intact mice and mice pretreated with 0.9% or 8.5% NaCl, respectively; and 6, 12, and 10 for NE in intact mice and mice pretreated with 0.9% or 8.5% NaCl, respectively. *p< 0.05, statistically significant difference between the selected groups.

The concentrations of L-DOPA in SON samples, normalized to protein, do not differ in all of the studied mice, including both intact animals and those 10 hours after intraperitoneal administration of 0.9% NaCl or 8.5% NaCl (**Figure 4**). In intact mice, the concentration of dopamine in the SON is approximately 4 times lower than the concentration of norepinephrine. In mice administered 0.9% or 8.5% NaCl, protein-normalized dopamine concentrations increased to the same extent in both cases – more than 2-fold; protein-normalized norepinephrine concentrations also increased, but to a greater extent – more than 3-fold (**Figure 4**).

### 3.3 Tyrosine hydroxylase in neurons of the supraoptic nucleus in mice after intraperitoneal administration of 0.9% NaCl or 8.5% NaCl and in neurons of the substantia nigra in intact mice according to Western blotting analysis using polyclonal and monoclonal antibodies

**Figure 5:**
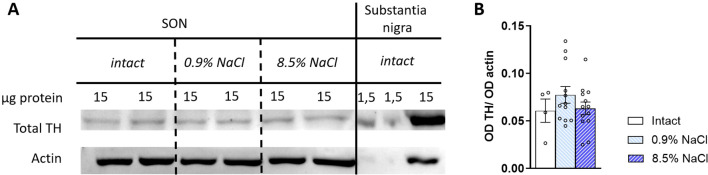
Tyrosine hydroxylase (TH) measured by western blotting in the supraoptic nucleus (SON) in 8- to 10-week-old C57BL/6 mice intact (n = 4) and 10 hours after intraperitoneal administration of 0.9% NaCl (n = 12) or 8.5% NaCl (n = 13) and substantia nigra (SN) in 8- to 10-week-old C57BL/6 mice (n = 2). **(A)** Representative image of the membrane immunostained for TH using polyclonal antibodies and for β-actin using monoclonal antibodies in SON and SN samples; **(B)** Optical density (OD) of TH-immunopositive material, normalized to the optical density of β-actin-immunopositive material in the SON. One-way Anova was used, assuming p<0.05 as a significant difference. The data are presented as mean ± SEM with individual date (dots).

The TH content, detected using polyclonal antibodies, in the SONs of mice 10 hours after intraperitoneal administration of 0.9% NaCl did not differ from that of mice after administration of 8.5% NaCl (p=0.3588) (**Figure 5**).

**Figure 6:**
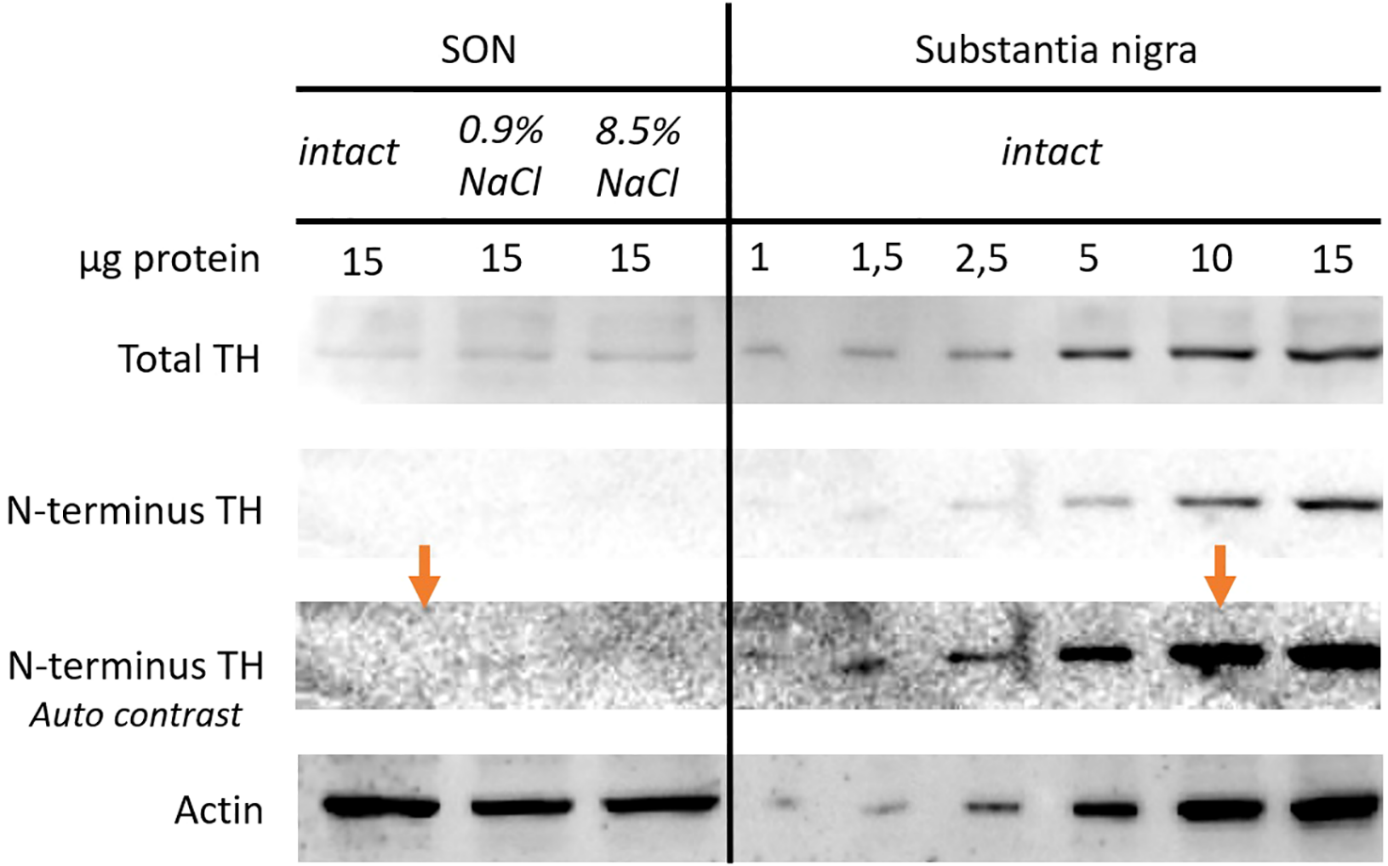
Representative images of western blotting membranes immunostained for tyrosine hydroxylase (TH) using polyclonal antibodies, for the N-terminus of the TH molecule using monoclonal antibodies, and for ß-actin in supraoptic nucleus (SON) of 8- to 10-week-old C57BL/6 mice - intact mice (n = 6) and mice 10 hours after intraperitoneal administration of 0.9% NaCl (n = 12) or 8.5% NaCl (n = 13), as well as in the substantia nigra of intact mice (n = 3). Sample loading for SON was 15 µg and for the substantia nigra – 1 to 15 µg of total protein on each line. For membrane stained with monoclonal antibodies for TH N-terminus, the result of auto-contrasting of the original image (orange arrows) is also shown.

Using monoclonal antibodies to the N-terminus of the TH molecule, the enzyme was detected in the substantia nigra of intact mice. In contrast to the substantia nigra, TH could not be detected with the same antibodies in the SON of osmotically stimulated mice (**Figure 6**).

## 4 Discussion

The development of a technique for double immunocytochemical labeling of TH and aromatic L-amino acid decarboxylase, the enzymes of dopamine synthesis, in the late 1980s (13, 31), has made it possible to show that, along with dopaminergic neurons expressing both enzymes of dopamine synthesis, there are neurons in the brain expressing only one of the enzymes, most often TH (12). It has turned out that monoenzymatic TH-containing neurons are localized mainly in the arcuate nucleus, periventricular nucleus, and the so-called magnocellular nuclei of the hypothalamus (11, 32, 33). Moreover, it has been shown that in most hypothalamic nuclei, with the exception of magnocellular nuclei, TH in monoenzymatic neurons is an enzyme that converts L-tyrosine to L-DOPA (12). However, repeated attempts to obtain direct evidence that TH in magnocellular neurons is an enzyme have been unsuccessful (17). Therefore, in the present study, we have returned to this issue, aiming to test the hypothesis that TH in magnocellular neurons in mice as an enzyme is capable of converting L-tyrosine to L-DOPA.

First, using LMD followed by real-time PCR, we showed that VP-immunopositive neurons of the SON express *Avp* and *Th*, but not *Gch1*, encoding guanosine triphosphate cyclohydrolase 1 (GCH1), the first enzyme in the synthesis of tetrahydrobiopterin (BH4). The latter is involved as a cofactor in the regulation of TH enzymatic activity and thus the conversion of L-tyrosine to L-DOPA (34). The absence of GCH1 in SON neurons is a strong argument against our hypothesis that TH in these neurons has enzymatic activity, providing L-DOPA synthesis from L-tyrosine. However, it cannot be ruled out that the PCR we used was not sensitive enough to detect GCH1 mRNA. This idea is indirectly supported by the fact that both GCH1 mRNA and the enzyme itself were detected in human SON neurons (24). Interestingly, in the human SON, the amount of GCH1 mRNA correlates with the intensity of TH immunostaining (24). However, GCH1 protein was not detected in SON in humans by Nagatsu et al. (25) and in rats by Marsais and Calas (17) did not detect it in the SON of rats, which supports the assumption that not only the expression of the GCH1 gene but also the synthesis of this enzyme are at the limit or even beyond the resolution of PCR and immunocytochemistry, respectively (24).

In the next study, we attempted to test the hypothesis that TH in SON neurons has enzymatic activity by assessing the yield of L-DOPA. For this, we compared L-DOPA levels in SON samples obtained from mice, including both intact animals and those following intraperitoneal injections of 0.9% NaCl or 8.5% NaCl, with quite different intraneuronal levels of TH (26). If our hypothesis that TH in SON neurons does have enzymatic activity is correct, then the level of L-DOPA we measured in SON tissue should increase as intraneuronal TH content increases. In other words, L-DOPA levels should be higher in mice after intraperitoneal administration of 0.9% NaCl compared with intact animals, and these levels should be even higher in mice that were intraperitoneally administered 8.5% NaCl. Surprisingly, the concentration of L-DOPA in the SON of mice in all three groups studied was the same and very low. This means that the concentration (yield) of L-DOPA does not depend on the content of TH in neurons of the SON, and therefore TH most probably does not have enzymatic activity in these neurons. In this case, the measured small amount of L-DOPA should be an intermediate product of catecholamine synthesis in catecholaminergic afferent fibers (15, 35, 36).

The results of our study are in good agreement with most previous studies, in which L-DOPA was not detected by immunocytochemistry in SON neurons, in contrast to neurons containing only TH in some other parts of the brain (16, 17, 24, 37–39). However, the lack of L-DOPA accumulation with increasing intraneuronal TH content in the SON observed in our study may be due not only to the fact that TH does not have enzymatic activity, but also due to a number of other reasons. First, the sensitivity of the high-performance liquid chromatography with electrochemical detection we used may be insufficient to detect small changes in the L-DOPA amount in neurons. Secondly, there is no convincing evidence that L-DOPA synthesized in monoenzymatic TH-containing neurons can be stored rather than released immediately after synthesis (12). Thirdly, in some studies, in addition to TH, mRNA for L-amino acid decarboxylase and the enzyme itself were found in SON neurons (24). It is well known that this enzyme quickly converts L-DOPA into dopamine in dopaminergic neurons (40). Finally, the number of L-amino acid decarboxylase-containing neurons in the SON is species-specific. Thus, tree shrews do have numerous L-amino acid decarboxylase-containing neurons (20), whereas such neurons are extremely rare in humans (24).

Our above data on the expression of *Th* and TH synthesis in SON neurons suggest that the TH molecule in these neurons may differ from that in dopaminergic neurons, since TH does not have enzymatic activity in these neurons. We tested this hypothesis in our further study of TH structure using Western blotting with polyclonal and monoclonal antibodies against the N-terminus of the TH molecule. We found that TH in SON neurons, in contrast to TH in dopaminergic neurons of the substantia nigra, could only be detected using polyclonal antibodies. This means that TH in SON neurons, in contrast to dopaminergic neurons (41, 42), could possibly not have the N-terminus motif, which is responsible for regulating the stability and enzymatic activity of TH (41, 42). The lack of TH N-terminus immunolabeling using Western blotting may be due to alternative mRNA splicing, protein degradation, or post-translational modifications that mask the corresponding epitope. However, according to Iwata et al. (43), alternative splicing of TH mRNA was not detected in mice (43). TH regulation is known to be complex due to the presence of multiple post-translational modifications that can interact with each other and affect enzymatic activity and protein stability. Thus, TH can be regulated by phosphorylation at serine residues at sites 8, 19, 31, and 40 (44), nitration at cysteine residue at site 279 (45), and O-GlcNAc glycosylation (46, 47). However, the glycosylation site of the enzyme has not been established.

The site most similar in composition to the epitope detected by our antibodies is the TH molecule is the phosphorylation site at position 19 of serine (pSer19). Although this phosphorylation does not directly affect the catalytic activity of TH, it may serve as a signal for its ubiquitin-proteasome degradation (48). Based on this, we propose that the lack of detection of the N-terminus of TH in the SON indicates degradation of this protein sequence. This assumption is indirectly supported by the rapid loss of TH-immunopositive neurons in the SON within 12–24 hours after salt loading (26). It has also been shown that the loss of 40 or more amino acids from the N-terminus significantly reduces TH activity (42). These findings suggest that the absence of the N-terminus in the TH molecule in SON neurons may cause the extremely low, if any, enzymatic activity of TH.

Thus, our data indicate that despite the expression of the *Th* and its translation into protein in SON neurons, this protein has no enzymatic activity, probably due to the absence of the N-terminus involved in the regulation of TH enzymatic activity in catecholaminergic neurons.

## Data Availability

The raw data supporting the conclusions of this article will be made available by the authors, without undue reservation.
