# Supplementary material for: Evidence that tyrosine hydroxylase does not have enzymatic activity in neurons of the supraoptic nucleus
Source: Front Endocrinol (Lausanne). 2026 Apr 28;17:1777453. doi: 10.3389/fendo.2026.1777453 (PMC13160761; doi:10.3389/fendo.2026.1777453)
Supplement: Supplementary file 1 [file DataSheet1.docx]

Supplementary Material

## Supplementary Figures


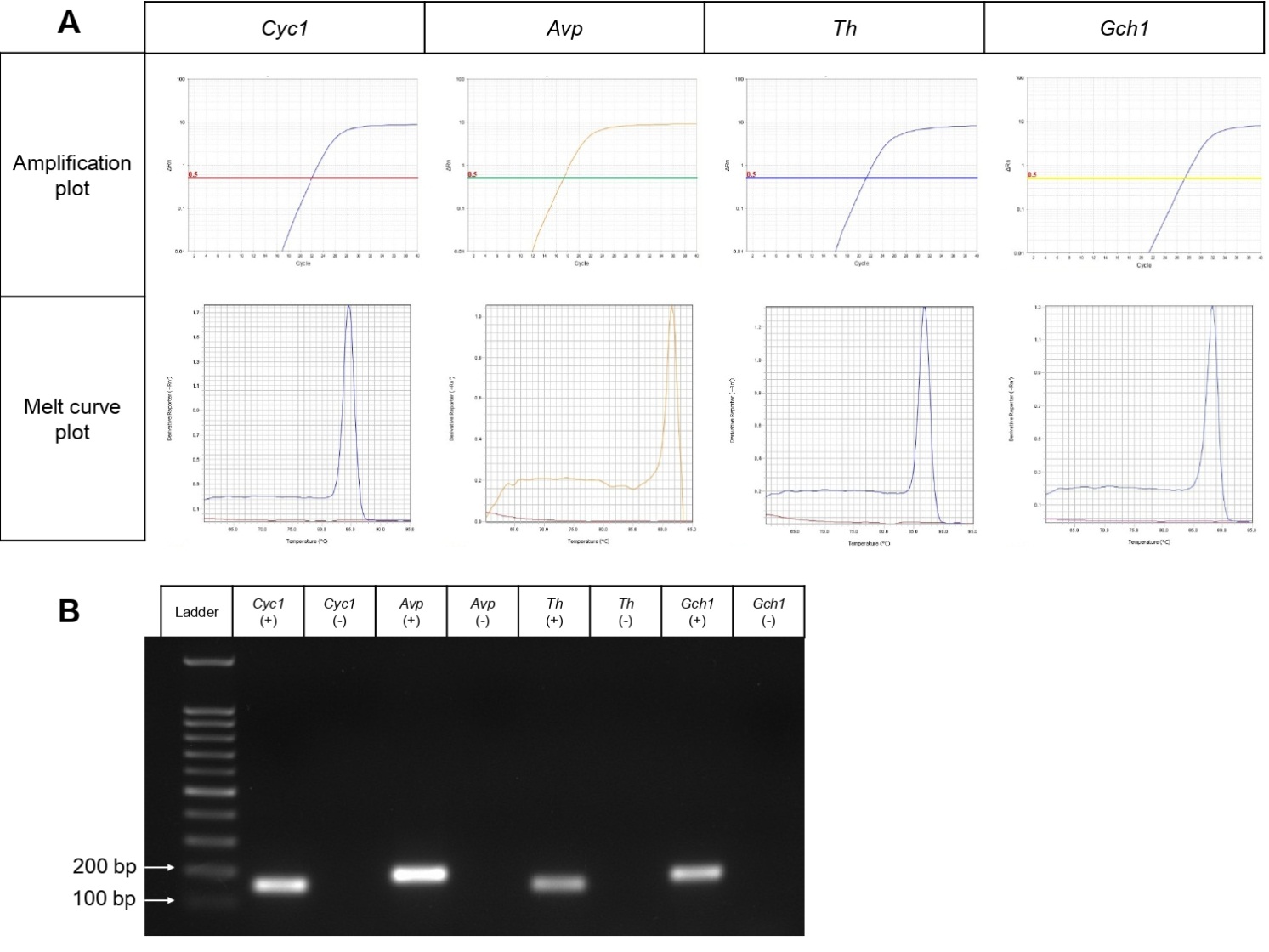


**Supplementary Figure 1.** Validation of primer pairs used for PCR. **(A)** Images of “Amplification plots” and “Melt curve plots” for positive controls (tissue sample whose cells are known to express the analyzed gene: substantia nigra tissue (*Cyc1*, *Th*, *Gch1*) or supraoptic nucleus tissue (*Avp*) and for negative controls (red lines: no template, cDNA was replaced with PCR grade water). **(B)** Agarose gel electrophoresis of PCR products (the amplification products were separated on a 1.5% agarose gel stained with ethidium bromide; Lane 1: molecular weight marker (100-1500 bp DNA ladder); Lanes 2, 4, 6, 8: positive controls for *Cyc1*, *Avp*, *Th*, and *Gch1*; Lanes 3, 5, 7, 9: negative controls for *Cyc1*, *Avp*, *Th*, and *Gch1* (no template). bp: base pair; + indicates positive controls; - indicates negative controls.
